# Chinese Herbal Medicines Might Improve the Long-Term Clinical Outcomes in Patients with Acute Coronary Syndrome after Percutaneous Coronary Intervention: Results of a Decision-Analytic Markov Model

**DOI:** 10.1155/2015/639267

**Published:** 2015-10-01

**Authors:** Shao-Li Wang, Cheng-Long Wang, Pei-Li Wang, Hao Xu, Ke-Ji Chen, Da-Zhuo Shi

**Affiliations:** ^1^Guang'anmen Hospital, China Academy of Chinese Medical Sciences, Beijing 100053, China; ^2^Xiyuan Hospital, China Academy of Chinese Medical Sciences, Beijing 100081, China

## Abstract

*Aims*. The priority of Chinese herbal medicines (CHMs) plus conventional treatment over conventional treatment alone for acute coronary syndrome (ACS) after percutaneous coronary intervention (PCI) was documented in the 5C trial (chictr.org number: ChiCTR-TRC-07000021). The study was designed to evaluate the 10-year effectiveness of CHMs plus conventional treatment versus conventional treatment alone with decision-analytic model for ACS after PCI. *Methods and Results*. We constructed a decision-analytic Markov model to compare additional CHMs for 6 months plus conventional treatment versus conventional treatment alone for ACS patients after PCI. Sources of data came from 5C trial and published reports. Outcomes were expressed in terms of quality-adjusted life years (QALYs). Sensitivity analyses were performed to test the robustness of the model. The model predicted that over the 10-year horizon the survival probability was 77.49% in patients with CHMs plus conventional treatment versus 77.29% in patients with conventional treatment alone. In combination with conventional treatment, 6-month CHMs might be associated with a gained 0.20% survival probability and 0.111 accumulated QALYs, respectively. *Conclusions*. The model suggested that treatment with CHMs, as an adjunctive therapy, in combination with conventional treatment for 6 months might improve the long-term clinical outcome in ACS patients after PCI.

## 1. Introduction

Percutaneous coronary intervention (PCI) as well as pharmacological treatments has significantly reduced but did not eliminate the risk of major adverse cardiovascular events (MACE) in ACS patients. It is reported that approximately 10%~18% of ACS survivors after PCI ultimately suffer a second myocardial infarction (MI), stroke, or cardiovascular death despite the availability of timely and appropriate treatments. With the raising concern of recurrent cardiovascular events in ACS patients undergoing primary PCI, it is necessary to substantiate the effectiveness and outcomes of adjunctive therapies, such as Chinese herbal medicines (CHMs) (e.g., xin mai tong capsule, Shexiang Baoxin Pill, and tongxinluo capsule) and acupuncture, when added to conventional medication.

CHMs have been widely used in clinical practice for thousands of years. Previously, we published the 1-year clinical outcomes of the 5C trial [1]. This multicenter, open-label, randomized controlled trial (chictr.org number: ChiCTR-TRC-07000021) showed that CHMs, Xinyue Capsule, and Fufang* Chuanxiong* Capsule, in combination with conventional treatment, further prevent 1-year occurrence of cardiovascular events in ACS patients after primary PCI without increasing risk of major bleeding, as compared with conventional treatment alone. Owing to the only 1-year follow-up period in this trial and other limited CHMs trial resources, the priority of CHMs in combination with conventional treatment over conventional treatment alone on long-term outcome of ACS patients has not been established.

Therefore, we constructed a decision-analysis Markov model to assess the effectiveness of CHMs plus conventional treatment versus conventional treatment alone in ACS patients after primary PCI. During 10 years, whether ACS patients after PCI may benefit from reducing the risk of MACE and increasing the quality of life (QOL) when treated by additional CHMs for 6 months, as an adjunctive therapy, in combination with conventional treatment, remains unclear.

## 2. Methods

### 2.1. Study Design

To capture the short- and long-term clinical outcomes in ACS patients after PCI receiving additional CHMs for 6 months plus conventional treatment versus conventional treatment alone, a decision-analytic Markov model was developed, by following generally accepted principles of design [2]. Referring to the model developed by previous studies [3–5], the model in the study comprises two components: the first part contains a decision tree which was in line with the period of the 5C trial (one year); the other part is that the subsequent events were modelled as a Markov structure with the potential for a recurrent event (subsequent years). The health outcomes modelled in the study were quality-adjusted life years (QALYs), which take into account both the quantity and QOL generated by the interventions. The model was based on the 5C trial's population, which included a broad spectrum of ACS patients, that is, ST-segment elevation MI, non-ST-elevation MI, and unstable angina (UA), who underwent successful PCI. The patients were randomized to receive additional CHMs for 6 months plus conventional treatment or conventional treatment alone. The aim of the modelling exercise was to adhere closely to the 5C trial and the model structure is based on the key clinical outcomes of 5C trial.

### 2.2. Model Structure

The 1-year decision tree was modelled based on the clinical outcomes in 5C trial. During the first year, the patients who received additional CHMs for 6 months plus conventional treatment or conventional treatment alone could suffer a nonfatal MI, a nonfatal stroke, a nonfatal UA, or death from all causes. Those patients who experienced no events were considered as event-free.

To simulate the long-term clinical outcomes in post-PCI ACS patients in 5C trial, the 1-year decision tree was extended to a long-term Markov model. A Markov model consists of a number of mutually exclusive and collectively exhaustive health states, usually named as Markov states, representing the disease progression process from entry to death or end of the time horizon of the analysis [6]. Disease progression or occurrence is modelled as transitions between states over time. In any given interval of time, referred to as a cycle or stage, a cohort member is in one and only one of the states. Patients who remain alive with an event spend 1 cycle in the first state of the corresponding event and then move on to the corresponding state for following cycles. The cycle length used in the model is 1 year.

**Figure 1:**
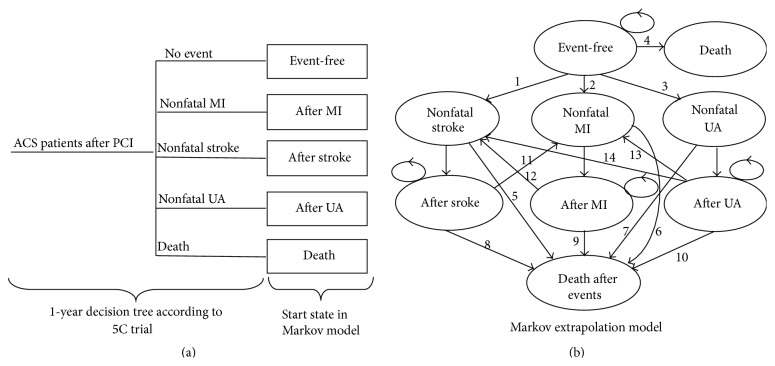
Two-component decision-analytic model structure. Part (a) is a decision tree representing the 5 clinical outcomes of the 5C trial during the 1-year period: event-free, nonfatal myocardial infarction (MI), nonfatal stroke, nonfatal unstable angina (UA), or all-cause death. Part (b) is long-term Markov model. (1) Risk of nonfatal stroke for event-free patients. (2) Risk of nonfatal MI for event-free patients. (3) Risk of nonfatal UA for event-free patients. (4) Mortality risk for event-free patients. (5) Mortality risk at the first year after a nonfatal stroke. (6) Mortality risk at the first year after a nonfatal MI. (7) Mortality risk at the first year after a nonfatal UA. (8) Mortality risk at second and subsequent years after a nonfatal stroke. (9) Mortality risk at second and subsequent years after a nonfatal MI. (10) Mortality risk at second and subsequent years after a nonfatal UA. (11) Risk of nonfatal MI for patients with stroke. (12) Risk of nonfatal stroke for patients with MI. (13) Risk of nonfatal MI for patients with UA. (14) Risk of nonfatal stroke for patients with UA.

In the study, the Markov model (Figure 1) had 8 health states, which were event-free, nonfatal AMI, post-MI, nonfatal stroke, poststroke, nonfatal UA, post-UA, and death (all-cause). Patients entered the Markov model based on the events in the 1-year decision tree. Patients who experienced no events during the first year in the decision tree entered the Markov model in the “event-free” state. These patients could suffer a fatal MI, stroke, or UA in every subsequent year and could also transit to a nonfatal MI, nonfatal stroke, or nonfatal UA state. Patients who suffered an MI, stroke, or UA in the 1-year decision tree entered the new MI, stoke, and new UA states in the Markov model, respectively. After 1 year in the new MI, new stroke, and new UA states, patients would transit to the corresponding postevent state. In each cycle, patients could experience a new MI, new stroke, and all-cause death or remain in a postevent state. The model assumed that patients could not enter the new UA state from the poststroke state and post-MI state due to the limitation of the relative data. Patients with a fatal event in the 1-year decision tree entered the Markov model as “dead,” the same as patients who died from the “no event” state. Patients who die in a nonfatal event state or postevent state pass to the dead postevents state.

The model was run up to a time horizon of 10 years. Half-cycle correction was performed in this study with assigning one-half of the state reward for simulated individuals starting in each state.

### 2.3. Transition Probabilities

Transition probabilities, which characterize how a cohort member may pass in successive cycles, vary over time and depend on patient characteristics but not on previous events as the model has no memory [7]. In 1-year decision tree, the probabilities of the patients experiencing nonfatal MI, stroke, or all-cause death were calculated from the data in 5C trial (Table 1).

In Markov model, as the intervention period of additional CHMs was only 6 months and no long-term data were available after the first year of treatment, we conservatively assumed that the transition probabilities were identical for patients receiving CHMs plus conventional treatment or conventional treatment alone for year 2 and onwards. And the only difference between the two treatment strategies was caused by the different distribution of patients in the different Markov states after the first year. To obtain the transition probabilities for the Markov model, data beyond the duration of the 5C trial were required. By following the methods used to derive the transition probabilities which had been previously published and validated in some trial-based economic analyses [8], the transition probabilities for nonfatal MI, nonfatal UA, nonfatal stroke, and death were also extrapolated on the basis of the available data drawn from the published reports. We conducted a literature search of PubMed, OVID, and the Cochrane Library websites for reaching results from cardiovascular trials from January 1980 up to December 2012. For reflecting the clinical outcomes in post-ACS patients, these probabilities were obtained from registry-based studies, analyses of randomized controlled trials, and systematic reviews which were preferred when available. The selection of the studies finally included in the model was performed in a nonsystematic way and conditioned on the adequacy of the data to the decision problem [9]. When necessary, reported and calculated rates from published reports were converted to probabilities for use in the model with the assumption of a constant hazard over time [10]. Details of the data sources and the transition probabilities are summarized in Table 2.

### 2.4. Utility Values

The outcomes of each treatment strategy were quantified in terms of QALYs over a 10-year horizon, as noted previously. To calculate QALYs, the utility weights were multiplied by the duration in each health state. An annual utility was assigned for each health state in the study.

Utility values describe the health-related QOL correlated with different health states on a scale of zero to one, where zero and one represent death and best imaginable health, respectively. The baseline utility values for patients of event-free during 1-year follow-up in CHM plus conventional treatment arm and conventional treatment alone arm were taken from 5C trial, in which health-related QOL was assessed at 1 year after PCI using EuroQol (EQ-5D). EQ-5D scores were derived using Japanese population tariff values [11]. Due to lack of the local utility values for patients with nonfatal AMI, nonfatal stroke, or nonfatal UA, the values proposed by published studies in the literatures were applied in the present analysis [12]. We calculated the disutility values by taking the difference in health-related QOL values between a patient with and without an event based on the method reported by Bagust et al. [13] and Chaplin et al. [14]. Patients experiencing an event (AMI, stroke, or UA) were assigned disutility weights to take into account the one-off decrease in their health status due to the event. For patients experiencing a MI, UA, or stroke, we attributed a disutility of 0.127, 0.117, and 0.139, respectively, at the time of the occurrence of an event until end of follow-up, which was obtained from a previous published study [15]. The baseline utility values used for each health states in the model as well as the ranges used within the sensitivity analyses are presented in Table 3.

### 2.5. Analytic Method and Univariate Sensitivity Analysis

For the 2 strategies, CHMs plus conventional treatment versus conventional treatment alone, we calculated QALYs and considered the strategy associated with a higher value to be preferred. Since our model was based on a number of assumptions and weighted average of published literature-derived probabilities, we performed univariate sensitivity analyses, in which we allowed any one of the variables of the model to vary at a time according to its estimates range, to determine whether and how plausible parameters in these assumptions and risks would alter our findings [10].

The Markov model was designed and all analyses were performed with TreeAge Pro Suite 2011 software package.

## 3. Results

### 3.1. Base-Case Analysis

**Figure 2:**
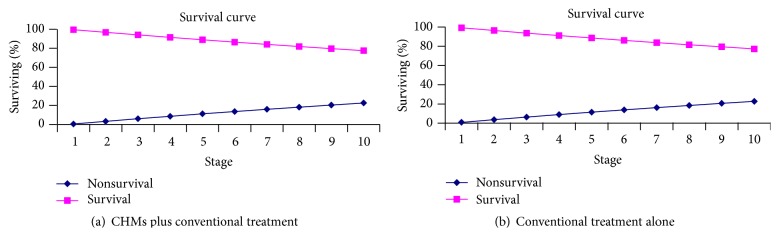
Survival curve.

The Markov model predicted that the discounted survival was higher in the CHMs plus conventional treatment arm when compared with the conventional treatment alone arm by 0.20% survival probability. The survival probability over the 10-year horizon was 77.49% in the CHMs plus conventional treatment arm and 77.29% in the conventional treatment alone arm, respectively (Figure 2).

In a cohort of 1000 patients over 10 years, the CHMs plus conventional treatment, compared with conventional treatment alone, would gain 22 patients remaining event-free, prevent 7, 9, and 5 patients further suffering from nonfatal MI, nonfatal UA, and nonfatal stroke, respectively, and avoid 20 patients dying from all causes.

**Figure 3:**
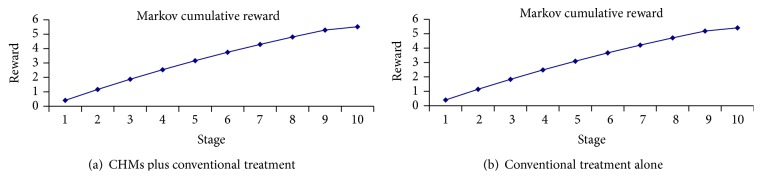
Cumulative QALYs over 10-year horizon.

The model predicted that participants after PCI who received CHMs plus conventional treatment would live an average of 0.405 discounted QALYs in 1-year and 5.519 discounted accumulated QALYs over 10-year horizon. And those who received conventional treatment alone would live an average of 0.396 QALYs in 1-year and 5.408 QALYs over 10-year horizon (Figure 3). Comparing with conventional treatment alone, CHMs plus conventional treatment would save 0.009 QALYs in 1 year and 0.111 QALYs for the time horizon of 10 years.

### 3.2. Sensitivity Analyses

The priority of CHMs plus conventional treatment over conventional treatment alone for all the parameters was considered in the univariate sensitivity analysis. The analysis showed that changes of every input parameter had no impact on the interpretation of the results. Thus, CHMs plus conventional treatment remained a dominant therapy over a broad range of the input parameters. The 5 most sensitive input parameters were annual mortality risk for event-free patients, annual risk of nonfatal UA for event-free patients, annual risk of nonfatal stroke for event-free patients, annual risk of nonfatal MI for event-free patients, and disutility of UA. The annual mortality risk for event-free patients (varied from 0.014 to 0.033) had the largest influence on the QALYs (5.505 to 5.532 for patients in the CHMs plus conventional treatment arm versus 5.393 to 5.423 for patients in the conventional treatment alone arm).

## 4. Discussion

In the present study, a two-component decision-analytic model approach was used to predict the short- and long-term effectiveness of CHMs plus conventional treatment and conventional treatment alone in the treatment of ACS after PCI. The results showed that CHMs plus conventional treatment would reduce the risk of death in ACS participants after PCI over 10-year period compared to conventional treatment alone, as well as the risk of nonfatal MI, nonfatal stroke, and nonfatal UA. Comparing with conventional treatment alone, CHMs plus conventional treatment would save 0.111 QALYs for the time horizon of 10 years. Given a cohort of 1000 patients over 10 years, the CHMs plus conventional treatment, compared with conventional treatment alone, would gain 22 patients benefit from no events and prevent 20 patients from all-cause death.

As far as we are concerned, this is the first study that has accounted for QOL and generated QALYs in estimating the long-term effectiveness of CHMs plus conventional treatment in ACS patients after PCI compared with conventional treatment alone. The study showed that the estimated gain with CHMs plus conventional treatment, compared with conventional treatment alone, was accrued due to an increase in survival probability and QOL as well. And the reduction in mortality and the increase in remaining event-free were the majority contributors to the survival probability. The favorable effectiveness of CHMs plus conventional treatment in our analysis was supported by earlier clinical studies, showing that CHMs plus conventional treatment were expected to reduce the risk of MI and improve myocardial reperfusion after PCI in patients with AMI during 3 months [16, 17], as well as playing roles in decreasing recurrent angina in patients with coronary heart disease [18, 19]. It should be noted that our findings extended the previous studies and dynamically analyzed the favorable effectiveness of CHMs plus conventional treatment over conventional treatment alone in the 10-year period, indicating that CHMs plus conventional treatment might be an adjunctive therapy in further improving the long-term clinical prognosis in patients with ACS after PCI. Since there was no study regarding long-term QALYs among ACS patients after PCI up to now in China, which used CHMs plus conventional treatment, more evidences were needed in the future to support the estimation of our study.

In the present study, the input parameters were derived from 5C trial and medical literatures. Over the first year, transition probabilities and baseline utility for patients in CHMs plus conventional treatment and conventional treatment alone arms were mainly taken from 5C trial. For some parameters, however, no data in 5C trial were available, so we used international data instead. In the absence of long-term data after 1 year (i.e., beyond the duration of 5C trial), the transition probabilities were obtained based on the collected data from registry-based studies, analyses of randomized controlled trials, and systematic reviews. Additionally, there were no previous published evidences of disutility values for patients experiencing MI, UA, or stroke after PCI in China; we used data from a published study [15] to perform the analysis as well.

In 5C trial, the intervention period of CHMs plus conventional treatment was only 6 months and there was no intervention difference in the subsequent 6 months between CHMs plus conventional treatment and conventional treatment alone. Our model made the conservative assumption that there was no incremental clinical benefit from CHMs plus conventional treatment versus conventional treatment alone beyond the first year of treatment; that is, the benefits of CHMs only worked in 6 months and beyond 1 year the transition probabilities were identical for both treatment arms. Advantages of using the decision-analytic model approach in long-term effectiveness evaluation are to be able to extend analyses beyond trial durations, to integrate data from a variety of sources, and to be able to explore the impact of the therapy in various treatment settings [20, 21]. The assumptions in the model, however, may not necessarily hold true. And we do not expect these assumptions to have a major influence on our results of the present study. Since further uncertainty arises through methodological and modeling structure uncertainty, which can be addressed with univariate sensitivity analysis [22], we performed univariate sensitivity analyses on various parameters and assumptions to assess the rigour of the assumptions on the effectiveness estimation. The analysis showed that our results are robust to very wide variations in model inputs.

Our study has several limitations. First, the study was performed based on a decision-analytic Markov model which was a simplification of reality, where probability data of outcomes occurred in another population and different scenarios from that of 5C trial were inevitably employed, although we used a resource estimate adapted to our reality [23]. The data sources about probabilities beyond 1 year were partly driven by the clinical event rates observed in the patients with NSTE-ACS or STEMI patients, while the population in 5C trial were combined NSTE-ACS patients and STEMI patients together. Even if GUSTO-IIb trial showed that the mortality rate at 30 days was greater among patients with ST-segment elevation than among those without ST elevation, this difference narrowed at 6 months and disappeared at 1 year [24], and the study reported by Singh et al. [25] also demonstrated that patients with STEMI and NSTEMI experienced similar outcomes; it was still difficult to exactly match the patients recruited in 5C trial. Somewhat, transition probabilities in our study were measured with some degree of error. Secondly, the study was supposed to predict the whole spectrum of consequences of therapy with a 10-year horizon, which requires making a series of difficult-to-demonstrate assumptions [9]. On the basis of the data currently available, our study might conservatively estimate the effectiveness of CHMs plus conventional treatment.

## 5. Conclusion

On the basis of the decision-analytic Markov model, the analysis suggested that treatment with CHMs, as an adjunctive therapy, in combination with conventional treatment for 6 months might improve the long-term clinical outcome in ACS patients after PCI. However, the larger long-term clinical trials are needed to prove the long-term effectiveness of CHMs plus conventional treatment in the treatment of ACS after PCI in the future.

## Figures and Tables

**Table 1:** Model parameters (1-year decision-analysis model).

Variables	Probability	
ACS after PCI	CHMs plus conventional treatment	Conventional treatment alone
Nonfatal AMI	0.005 (0, 0.0119)	0.0175 (0.0047, 0.0303)
Nonfatal stroke	0.0074 (0, 0.0158)	0.0150 (0.0031, 0.0269)
Nonfatal UA	0.0149 (0.0031, 0.0267)	0.0399 (0.0207, 0.0591)
Death	0.0050 (0, 0.0119)	0.0075 (0, 0.0159)

ACS: acute coronary syndrome; PCI: percutaneous coronary intervention; AMI: acute myocardial infarction; CHMs: Chinese herbal medicines; UA: unstable angina.

**Table 2:** Transition probabilities among health states in the long-term Markov model.

Variables	Baseline probability	Range	Source
Event-free followed by			
Nonfatal AMI	0.018	0.010–0.020	[26–35]
Nonfatal stroke	0.007	0.001–0.009	[36–38]
Nonfatal UA	0.03	0.02–0.05	[39–41]
Death	0.027	0.014–0.033	[26–35]
Post-MI followed by			
Death, 1st year	0.039	0.008–0.076	[33, 42–56]
Death, after 1st year	0.021	0.003–0.027	[33, 42, 45–53, 56]
Nonfatal AMI, 1st year	0.024	0.002–0.060	[33, 42–56]
Nonfatal AMI, after 1st year	0.018	0.001–0.008	[33, 42, 44–53, 56]
Nonfatal stroke, 1st year	0.010	0.0024–0.024	[33, 43, 44, 57–61]
Nonfatal stroke, after 1st year	0.007	0.0008–0.022	[58, 59]
Post-UA followed by			
Death, 1st year	0.034	0.012–0.050	[30, 32, 33]
Death, after 1st year	0.020	0.016–0.028	[30, 32, 33]
Nonfatal AMI, 1st year	0.036	0.01–0.05	[30, 33, 59, 62]
Nonfatal AMI, after 1st year	0.011	0.010–0.063	[30, 33, 59, 62]
Nonfatal stroke, 1st year	0.018	0.014–0.023	[33, 62]
Nonfatal stroke, after 1st year	0.008	0.006–0.01	[33, 62]
Post stroke followed by			
Death, 1st year	0.115	0.066–0.189	[58, 62–69]
Death, after 1st year	0.035	0.016–0.061	[58, 62, 64–67, 69]
Nonfatal AMI, 1st year	0.003	0.002–0.006	[58, 59]
Nonfatal AMI, after 1st year	0.004	0.002–0.006	[58, 59]
Nonfatal stroke, 1st year	0.128	0.064–0.189	[68, 70, 71]
Nonfatal stroke, after 1st year	0.040	0.030–0.080	[71, 72]
Rate of age-related MACE (OR/10 years)	0.5	0.33–0.87	[28, 30, 31, 39, 69]

AMI: acute myocardial infarction; UA: unstable angina; MACE: major adverse cardiovascular events.

**Table 3:** Estimated utilities and disutilities.

Events	Base-case value	Range	Source
Event-free			
CHMs plus conventional treatment	0.818	0.418 to 0.848	5C trial
Conventional treatment alone	0.809	0.252 to 0.848	5C trial
Disutilities (QALYs)			
Nonfatal AMI	0.127	0.108 to 0.147	[15]
Nonfatal Stroke	0.139	0.118 to 0.160	[15]
Nonfatal UA	0.117	0.100 to 0.135	[15]
Death	0		

CHMs: Chinese herbal medicines; AMI: acute myocardial infarction; UA: unstable angina.

## References

[1] Wang S.-L., Wang C.-L., Wang P.-L. (2013). Combination of Chinese herbal medicines and conventional treatment versus conventional treatment alone in patients with acute coronary syndrome after percutaneous coronary intervention (5C trial): an open-label randomized controlled, multicenter study. *Evidence-Based Complementary and Alternative Medicine*.

[2] Weinstein M. C., Fineberg H. V. (1980). *Clinical Decision Analysis*.

[3] Nikolic E., Janzon M., Hauch O., Wallentin L., Henriksson M. (2013). Cost-effectiveness of treating acute coronary syndrome patients with ticagrelor for 12 months: results from the PLATO study. *European Heart Journal*.

[4] Chin C. T., Mellstrom C., Chua T. S., Matchar D. B. (2013). Lifetime cost-effectiveness analysis of ticagrelor in patients with acute coronary syndromes based on the PLATO trial: a Singapore healthcare perspective. *Singapore Medical Journal*.

[5] Theidel U., Asseburg C., Giannitsis E., Katus H. (2013). Cost-effectiveness of ticagrelor versus clopidogrel for the prevention of atherothrombotic events in adult patients with acute coronary syndrome in Germany. *Clinical Research in Cardiology*.

[6] Tseng M.-C., Chang K.-C. (2004). Cost-effectiveness analysis of tissue plasminogen activator for acute ischemic stroke: a comparative review. *Acta Neurologica Taiwanica*.

[7] Lindgren P., Jönsson B., Yusuf S. (2004). Cost-effectiveness of clopidogrel in acute coronary syndromes in Sweden: a long-term model based on the cure trial. *Journal of Internal Medicine*.

[8] Gasche D., Ulle T., Meier B., Greiner R.-A. (2013). Cost-effectiveness of ticagrelor and generic clopidogrel in patients with acute coronary syndrome in Switzerland. *Swiss Medical Weekly*.

[9] Latour-Perez J., De-Miguel-Balsa E. (2009). Cost effectiveness of fondaparinux in non-ST-elevation acute coronary syndrome. *PharmacoEconomics*.

[10] Garg P., Cohen D. J., Gaziano T., Mauri L. (2008). Balancing the risks of restenosis and stent thrombosis in bare-metal versus drug-eluting stents. results of a decision analytic model. *Journal of the American College of Cardiology*.

[11] Li M. H., Luo N. (2009). Introduction on the application of the European five-dimensional health scale. *Chinese Journal of Pharmaceutical Economics*.

[12] Kourlaba G., Maniadakis N., Andrikopoulos G., Vardas P. (2014). Economic evaluation of rivaroxaban in stroke prevention for patients with atrial fibrillation in Greece. *Cost Effectiveness and Resource Allocation*.

[13] Bagust A., Grayson A. D., Palmer N. D., Perry R. A., Walley T. (2006). Cost effectiveness of drug eluting coronary artery stenting in a UK setting: cost-utility study. *Heart*.

[14] Chaplin S., Scuffham P. A., Alon M. (2004). Secondary prevention after PCI: the cost-effectiveness of statin therapy in the Netherlands. *Netherlands Heart Journal*.

[15] Wagner M., Goetghebeur M., Merikle E., Pandya A., Chu P., Taylor D. C. A. (2009). Cost-effectiveness of intensive lipid lowering therapy with 80 mg of atorvastatin, versus 10 mg of atorvastatin, for secondary prevention of cardiovascular disease in Canada. *Canadian Journal of Clinical Pharmacology*.

[16] Li Y., Jin M., Qiu S. (2009). Effect of Chinese herbal medicine for Benefiting Qi and Nourishing Yin to promote blood circulation on ventricular wall motion of AMI patients after revascularization. *Chinese Journal of Integrated Traditional and Western Medicine*.

[17] Qiu S., Jin M., Zhu T. (2009). Effect of replenishing Qi and nourishing Yin to promote the blood circulation on 103 patients with acute myocardial infarction after reperfusion. *Journal of Capital Medical University*.

[18] Sheng G., Niu D., Wu S. (2009). Influence of complex Chuanxiong capsule on the blood fat of coronary and the heart function. *China Journal of Modern Medicine*.

[19] Zhang W. (2011). Clinical study of Fufang Chuanxiong capsule on Angina pectoris. *Medical Innovation of China*.

[20] Buxton M. J., Drummond M. F., Van Hout B. A. (1997). Modelling in economic evaluation: an unavoidable fact of life. *Health Economics*.

[21] Chambers M., Hutton J., Gladman J. (1999). Cost-effectiveness analysis of antiplatelet therapy in the prevention of recurrent stroke in the UK. Aspirin, dipyridamole and aspirin-dipyridamole. *PharmacoEconomics*.

[22] Bischof M., Briel M., Bucher H. C., Nordmann A. (2009). Cost-effectiveness of drug-eluting stents in a US medicare setting: a cost-utility analysis with 3-year clinical follow-up data. *Value in Health*.

[23] Araújo D. V., Tura B. R., Brasileiro A. L., Neto H. L., Pavão A. L. B., Teich V. (2008). Cost-effectiveness of prehospital versus inhospital thrombolysis in acute myocardial infarction. *Arquivos Brasileiros de Cardiologia*.

[24] Armstrong P. W., Fu Y., Chang W.-C. (1998). Acute coronary syndromes in the GUSTO-IIb trial: prognostic insights and impact of recurrent ischemia. *Circulation*.

[25] Singh M., Reeder G. S., Jacobsen S. J., Weston S., Killian J., Roger V. L. (2002). Scores for post-myocardial infarction risk stratification in the community. *Circulation*.

[26] Ellis S. G., Stone G. W., Cox D. A. (2009). Long-term safety and efficacy with paclitaxel-eluting stents 5-year final results of the TAXUS IV clinical trial (TAXUS IV-SR: treatment of de novo coronary disease using a single paclitaxel-eluting stent). *Journal of the American College of Cardiology: Cardiovascular Interventions*.

[27] Weisz G., Leon M. B., Holmes D. R. (2009). Five-year follow-up after sirolimus-eluting stent implantation: results of the SIRIUS (sirolimus-eluting stent in de-novo native coronary lesions) trial. *Journal of the American College of Cardiology*.

[28] Leon M. B., Allocco D. J., Dawkins K. D. (2009). Late clinical events after drug-eluting stents: the interplay between stent-related and natural history-driven events. *Journal of the American College of Cardiology: Cardiovascular Interventions*.

[29] Stone G. W., Moses J. W., Ellis S. G. (2007). Safety and efficacy of sirolimus- and paclitaxel-eluting coronary stents. *The New England Journal of Medicine*.

[30] Caixeta A., Leon M. B., Lansky A. J. (2009). 5-Year clinical outcomes after sirolimus-eluting stent implantation insights from a patient-level pooled analysis of 4 randomized trials comparing sirolimus-eluting stents with bare-metal stents. *Journal of the American College of Cardiology*.

[31] Fox K. A. A., Poole-Wilson P. A., Henderson R. A. (2002). Interventional versus conservative treatment for patients with unstable angina or non-ST-elevation myocardial infarction: the British Heart Foundation RITA 3 randomised trial. *The Lancet*.

[32] Simsek C., Magro M., Boersma E. (2010). The unrestricted use of sirolimus- and paclitaxel-eluting stents results in better clinical outcomes during 6-year follow-up than bare-metal stents an analysis of the RESEARCH (rapamycin-eluting stent evaluated at rotterdam cardiology hospital) and T-SEARCH (taxus-stent evaluated at rotterdam cardiology hospital) registries. *Journal of the American College of Cardiology: Cardiovascular Interventions*.

[33] Kawaguchi R., Kimura T., Morimoto T. (2010). Safety and efficacy of sirolimus-eluting stent implantation in patients with acute coronary syndrome in the real world. *American Journal of Cardiology*.

[34] Mauri L., Massaro J. M., Jiang S. (2010). Long-term clinical outcomes with zotarolimus-eluting versus bare-metal coronary stents. *Journal of the American College of Cardiology: Cardiovascular Interventions*.

[35] Zhang Q., Xu B., Yang Y.-J. (2008). Long term efficacy and safety of Chinese made sirolimus eluting stents: results, including off label usage, from two centres over three years. *Chinese Medical Journal*.

[36] Eisenstein E. L., Wijns W., Fajadet J. (2009). Long-term clinical and economic analysis of the endeavor drug-eluting stent versus the driver bare-metal stent 4-year results from the endeavor II trial, (randomized controlled trial to evaluate the safety and efficacy of the medtronic AVE ABT-578 eluting driver coronary stent in de novo native coronary artery lesions). *Journal of the American College of Cardiology: Cardiovascular Interventions*.

[37] Rosen V. M., Taylor D. C., Parekh H. (2010). Cost effectiveness of intensive lipid-lowering treatment for patients with congestive heart failure and coronary heart disease in the US. *Pharmacoeconomics*.

[38] Heeg B. M. S., Peters R. J. G., Botteman M. (2007). Long-term clopidogrel therapy in patients receiving percutaneous coronary intervention. *Pharmacoeconomics*.

[39] Boden W. E., O'Rourke R. A., Teo K. K. (2007). Optimal medical therapy with or without PCI for stable coronary disease. *The New England Journal of Medicine*.

[40] Henderson R. A., Pocock S. J., Clayton T. C. (2003). Seven-year outcome in the RITA-2 trial: coronary angioplasty versus medical therapy. *Journal of the American College of Cardiology*.

[41] Dagenais G. R., Lu J., Faxon D. P. (2011). Effects of optimal medical treatment with or without coronary revascularization on angina and subsequent revascularizations in patients with type 2 diabetes mellitus and stable ischemic heart disease. *Circulation*.

[42] Ko D. T., Chiu M., Guo H. (2009). Safety and effectiveness of drug-eluting and bare-metal stents for patients with off- and on-label indications. *Journal of the American College of Cardiology*.

[43] Sim D. S., Jeong M. H., Ahn Y. (2011). Effectiveness of drug-eluting stents versus bare-metal stents in large coronary arteries in patients with acute myocardial infarction. *Journal of Korean Medical Science*.

[44] Kaltoft A., Kelbæk H., Thuesen L. (2010). Long-term outcome after drug-eluting versus bare-metal stent implantation in patients with ST-segment elevation myocardial infarction: 3-year follow-up of the randomized DEDICATION (drug elution and distal protection in acute myocardial infarction) trial. *Journal of the American College of Cardiology*.

[45] Di Lorenzo E., De Luca G., Sauro R. (2009). The PASEO (PaclitAxel or sirolimus-eluting stent versus bare metal stent in primary angioplasty) randomized trial. *Journal of the American College of Cardiology: Cardiovascular Interventions*.

[46] Valgimigli M., Campo G., Arcozzi C. (2007). Two-year clinical follow-up after sirolimus-eluting versus bare-metal stent implantation assisted by systematic glycoprotein IIb/IIIa Inhibitor Infusion in patients with myocardial infarction: results from the STRATEGY study. *Journal of the American College of Cardiology*.

[47] Violini R., Musto C., De Felice F. (2010). Maintenance of long-term clinical benefit with sirolimus-eluting stents in patients with ST-segment elevation myocardial infarction 3-year results of the SESAMI (sirolimus-eluting stent versus bare-metal stent in acute myocardial infarction) trial. *Journal of the American College of Cardiology*.

[48] Atary J. Z., van der Hoeven B. L., Liem S. S. (2010). Three-year outcome of sirolimus-eluting versus bare-metal stents for the treatment of ST-segment elevation myocardial infarction (from the MISSION! Intervention study). *American Journal of Cardiology*.

[49] Spaulding C., Teiger E., Commeau P. (2011). Four-year follow-up of TYPHOON (trial to assess the use of the CYPHer sirolimus-eluting coronary stent in acute myocardial infarction treated with BallOON angioplasty). *Journal of the American College of Cardiology: Cardiovascular Interventions*.

[50] Vink M. A., Dirksen M. T., Suttorp M. J. (2011). 5-Year follow-up after primary percutaneous coronary intervention with a paclitaxel-eluting stent versus a bare-metal stent in acute ST-segment elevation myocardial infarction: a follow-up study of the PASSION (paclitaxel-eluting versus conventional stent in myocardial infarction with ST-segment elevation) trial. *Journal of the American College of Cardiology: Cardiovascular Interventions*.

[51] Kim H.-S., Lee J.-H., Lee S.-W. (2011). Long-term safety and efficacy of sirolimus- vs. paclitaxel-eluting stent implantation for acute ST-elevation myocardial infarction: 3-year follow-up of the PROSIT trial. *International Journal of Cardiology*.

[52] Piccolo R., Cassese S., Galasso G. (2011). Long-term safety and efficacy of drug-eluting stents in patients with acute myocardial infarction: a meta-analysis of randomized trials. *Atherosclerosis*.

[53] Brar S. S., Leon M. B., Stone G. W. (2009). Use of drug-eluting stents in acute myocardial infarction: a systematic review and meta-analysis. *Journal of the American College of Cardiology*.

[54] Kastrati A., Dibra A., Spaulding C. (2007). Meta-analysis of randomized trials on drug-eluting stents vs. bare-metal stents in patients with acute myocardial infarction. *European Heart Journal*.

[55] Pan X.-H., Chen Y.-X., Xiang M.-X. (2010). A meta-analysis of randomized trials on clinical outcomes of paclitaxel-eluting stents versus bare-metal stents in ST-segment elevation myocardial infarction patients. *Journal of Zheijang University SCIENCE B (Biomedicine & Biotechnology)*.

[56] Mauri L., Silbaugh T. S., Garg P. (2008). Drug-eluting or bare-metal stents for acute myocardial infarction. *The New England Journal of Medicine*.

[57] Stone G. W., Lansky A. J., Pocock S. J. (2009). Paclitaxel-eluting stents versus bare-metal stents in acute myocardial infarction. *The New England Journal of Medicine*.

[58] Lamotte M., Annemans L., Evers T. (2006). A multi-country economic evaluation of low-dose aspirin in the primary prevention of cardiovascular disease. *Pharmacoeconomics*.

[59] Ward S., Jones M. L., Pandor A. (2007). A systematic review and economic evaluation of statins for the prevention of coronary events. *Health Technology Assessment*.

[60] Tanne D., Goldbourt U., Zion M. (1993). Frequency and prognosis of stroke/TIA among 4808 survivors of acute myocardial infarction. The SPRINT Study Group. *Stroke*.

[61] Pedersen T. R., Faergeman O., Kastelein J. J. P. (2005). High-dose atorvastatin vs usual-dose simvastatin for secondary prevention after myocardial infarction the IDEAL study: a randomized controlled trial. *Journal of the American Medical Association*.

[62] Lindgren P., Jönsson B., Yusuf S. (2004). Cost-effectiveness of clopidogrel in acute coronary syndromes in Sweden: a long-term model based on the cure trial. *Journal of Internal Medicine*.

[63] Wiviott S. D., Braunwald E., McCabe C. H. (2007). Prasugrel versus clopidogrel in patients with acute coronary syndromes. *The New England Journal of Medicine*.

[64] Chang K.-C., Lee H.-C., Tseng M.-C. (2010). Three-year survival after first-ever ischemic stroke is predicted by initial stroke severity: a hospital-based study. *Clinical Neurology and Neurosurgery*.

[65] Koton S., Tanne D., Green M. S. (2010). Mortality and predictors of death 1 month and 3 years after first-ever ischemic stroke: data from the first national acute stroke Israeli survey (NASIS 2004). *Neuroepidemiology*.

[66] Han D. S., Pan S. L., Chen S. Y. (2008). Predictors of long-term survival after stroke in Taiwan. *Journal of Rehabilitation Medicine*.

[67] Bravata D. M., Ho S. Y., Brass L. M. (2003). Long-term mortality in cerebrovascular disease. *Stroke*.

[68] Petty G. W., Brown R. D., Whisnant J. P. (2000). Ischemic stroke subtypes: a population-based study of functional outcome, survival, and recurrence. *Stroke*.

[69] Ringborg A., Lindgren P., Jönsson B. (2005). The cost-effectiveness of dual oral antiplatelet therapy following percutaneous coronary intervention: a Swedish analysis of the CREDO trial. *The European Journal of Health Economics*.

[70] Hardie K., Hankey G. J., Jamrozik K. (2004). Ten-year risk of first recurrent stroke and disability after first-ever stroke in the Perth Community Stroke study. *Stroke*.

[71] Hankey G. J. (2003). Long-term outcome after ischaemic stroke/transient ischaemic attack. *Cerebrovascular Diseases*.

[72] Hankey G. J., Jamrozik K., Broadhurst R. J. (1998). Long-term risk of first recurrent stroke in the Perth Community Stroke study. *Stroke*.

